# Graying arts access: crafting creative online programming to promote older adults' artistic engagement in and beyond pandemic time

**DOI:** 10.3389/fsoc.2024.1454143

**Published:** 2024-12-04

**Authors:** Jami McFarland, Carla Rice, Nadine Changfoot, Tara La Rose, Carmela Alfaro-Laganse, Suad Badri, Kathy Smith, Becky Katz

**Affiliations:** ^1^The Re•Vision Centre for Art and Social Justice, University of Guelph, Guelph, ON, Canada; ^2^School of Occupational Therapy, Western University, London, ON, Canada; ^3^Political Studies and Trent Centre for Aging & Society, Trent University, Peterborough, ON, Canada; ^4^School of Social Work, McMaster University, Hamilton, ON, Canada; ^5^School of Arts, Faculty of Humanities, McMaster University, Hamilton, ON, Canada; ^6^Ahfad University for Women, Omdurman, Sudan; ^7^Hamilton Artistic Inc., Hamilton, ON, Canada; ^8^Creative Age Network, London, ON, Canada; ^9^Creative Age Art Studio, Dorchester, ON, Canada; ^10^Independent Artist, Hamilton, ON, Canada

**Keywords:** aging, aging-disability nexus, arts access, remote access, COVID-19, underrepresented older adults

## Abstract

**Introduction:**

Declared a global pandemic by the World Health Organization (WHO) in March 2020, the COVID-19 virus and attendant patchwork of local, regional, and national government-initiated public health responses to it unexpectedly opened possibilities for greater access to culture for disabled and chronically ill people in ways that were unimagined in pre-pandemic times. During the “emergency” period of the pandemic, the fields of critical disability studies and aging studies independently demonstrated the importance and value of shifting to digital technologies for disabled people and older adults respectively; however, to date, little scholarship has considered the value of digital technologies for older adults aging with and into disabilities beyond pandemic time.

**Methods:**

Informed by the theoretical insights of scholarship exploring critical access and the aging-disability nexus, this paper draws from empirical data collected during Phase 2 of Direct[Message]: Digital Access to Artistic Engagement, a collaborative, community-based, arts-informed research project based in Southwestern Ontario (Canada). Drawing from 50 qualitative interviews with aging adults from un/under/represented communities, findings explore the intersections of older age and disability, including dynamics related to gender, sexuality, migration, size, race/ethnicity, and other differences, as these relate to access to and enjoyment of creative spaces before, during, and “after” the COVID-19 pandemic.

**Results:**

Results show that older adults aging with/into disabilities in Southwestern Ontario express an overwhelming desire and even urgent need to access interactive arts programming from the relatively safe spaces of their homes both within and outside pandemic time.

**Discussion:**

As the normative world pushed for a return to ableist normative life in 2022, a year marked by “severe” rates of the highly infectious Omicron variant and the loss of effective public measures, such as community masking and widely available testing, participants described the need for continued access to creative and social participation via remote options that sidestepped socially exclusive and physically inaccessible spaces. Findings indicate a need for increased investment in digital arts programming for older adults aging with/into disabilities.

## Introduction

Declared a global pandemic by the World Health Organization (WHO) in March 2020, the COVID-19 virus and attendant patchwork of local, regional, and national government-initiated public health responses to it unexpectedly opened possibilities for greater access to culture for disabled and chronically ill people in ways that were unimagined in pre-pandemic times (Barden et al., 2023; Brown, 2021; Introna, 2023; Rice et al., 2021). Because the pandemic posed a threat to coded-as-abled/healthy populations, “emergency” measures (Ellcessor, 2022) or temporary forms of access proliferated and “many disabled people noted that the pandemic made for a ‘cripping of the world'—where for perhaps the first time the vast majority of humanity ‘dwelled in disabled reality”' (Piepzna-Samarasinha, 2022a, n.p.; Croft et al., 2024; Ignagni et al., 2020; Keegan, 2020; Rice et al., 2024; Wong, 2022). For example, for millions of disabled and older people, orders to shelter-in-place or stay at home were “ironic” since confinement to the home has historically been the default reality for many disabled people, including older adults experiencing changes to hearing, sight, memory, and/or mobility; and loneliness and social isolation were, prior to the pandemic, viewed predominantly as a social problem affecting older communities (Clayton et al., 2023; Goggin and Ellis, 2020; O‘Sullivan et al., 2021). During the emergency period, however, such confinement was experienced by young, nondisabled, and healthy populations as a “new normal” or temporary reality that generated some promising yet *temporary* solidarity with disabled and aging communities (Nowakowski, 2023; Piepzna-Samarasinha, 2022b). Pandemic emergency responses necessitated the widespread adoption of digital technologies to support remote working, learning, and socializing, and marked a period that some critical disability studies scholars have described as “pandemic time” or “COVID time,” a “time that [for normative life] has to be endured rather than settled into” (Ignagni et al., 2020, para 9; Croft et al., 2024; Ellcessor, 2022).

At the beginning of the pandemic, some scholarly and public discourses framed emergency measures (e.g., restricting in-person interactions) as constituting a time of increased recognition of “crip” culture and access practices (e.g., digital/remote access), and thus ushering in a period of greater inclusion for disabled people—“crip,” a reclaimed pejorative term, conceptualizes disability not as dysfunction, but rather as possibility (Clare, 1999; Rice et al., 2021). During these same years, however, counter-discourses emerged to simultaneously frame emergency measures and the swift turn to the digital as instituting a time of intense isolation, loneliness, and exclusion—essentially, a time of no access to cultural or social life—for older adults, generally (Berg-Weger and Morley, 2020; Moore and Hancock, 2020; Reneland-Forsman, 2020; Seifert, 2020; Shan et al., 2020; Zapletal et al., 2023). The shift to virtually delivered services, while beneficial for some (e.g., young disabled and/or chronically ill communities), has highlighted a critical need to promote the digital engagement of older adults who disproportionately encounter unevenly felt “digital divides,” or barriers to digital/remote access that can compound the risk of experiencing social isolation in later life, and exponentially exacerbate crosscutting digital inequalities (Beaunoyer et al., 2020; Cosco et al., 2021; Losada-Baltar et al., 2021; Zheng and Walsham, 2021). Although access to and use of technology among older adults increased during the pandemic (Clayton et al., 2023; Murciano-Hueso et al., 2022; Sixsmith et al., 2022), especially among those with family members or caregivers who could assist with technology training and use, many older adults continue to face an array of barriers, including digital anxiety, fear of new technologies and cybercrime, low digital competencies, and a lack of digital skill-building opportunities (Kim et al., 2023; La Rose et al., 2022; Schlomann et al., 2020; Tomczyk et al., 2023). For multiply marginalized older adults who experience additional barriers to social participation, such as discrimination and cost, the inaccessibility of creative digital spaces has emerged as especially harmful (Jonsson et al., 2023). Such work has highlighted a critical need in aging studies to take seriously older adults' use of technology and the digital inequalities they experience, a problematic that the pandemic and emergency responses to it both accentuated and aggravated. Although numerous studies have demonstrated the role technology and the arts play in decreasing social isolation, establishing and sustaining social belonging, promoting lifelong learning, and improving the well-being of older adults (Castora-Blinkey et al., 2010; Cohen, 2006; Guthell and Heyman, 2016; Klimczuk, 2017; McFadden and Basting, 2010; Noice et al., 2014; Peine et al., 2021; Todd et al., 2017), few studies with a focus on aging have considered how cultural and artistic participation could be facilitated using digital technologies prior to the pandemic (La Rose et al., 2022).

In contrast, critical disability studies has demonstrated the value of digital technologies and environments before, during, and beyond pandemic time by evidencing the *political* significance of digital access to artistic engagement and creative participation for disability-identified communities (Cachia, 2023; Chandler, 2019; Chandler et al., 2018; Orsini and Kelly, 2016; Rice et al., 2015, 2016, 2018, 2024, 2023). Researching during pandemic emergency conditions, some critical disability scholars have uncovered and highlighted the creative possibilities and unanticipated benefits of digital and/or remote options. This includes the Narratives of Neurodiversity Network, a neurodivergent academic, creative, and educator collective, who explain:

As the pandemic mainstreamed remote collaboration to an unprecedented level, we realized the possibilities an online space could offer neurodivergent individuals outside the oppressive and pathologizing structures of societal institutions, including the classroom, the courtroom, the psychiatrist's chair, and the academy. (Betts et al., 2023, p. 64)

Indeed, remote access, according to Johnson et al. (2024), comprises “an example of crip technoscience and a crip ritual—a transformative practice that is ‘repeated and reiterated within disability culture”' (p. 218). Remote access events or parties, whereby participants come together and interact in virtual space in ways that sidestep the inaccessible dynamics and/or harmful effects of gathering in-person, offer vivid illustrations of disabled community-building (Gotkin and Hamraie, 2024; Hamraie and williams, 2023; Johnson et al., 2024). As well, since the start of the pandemic, chronically ill and disabled people have depended on digital platforms (e.g., Twitter and Instagram) to find community, track COVID-19, make its symptoms and after-effects visible, tackle misinformation, and circulate life-saving resources (Callard, 2020). At the time of writing, social media accounts (e.g., DoNoHarm BC) and COVID trackers, such as the Canadian COVID-19 Hazard Index (COVID-19 Resources Canada, 2024), continue to circulate in online disability communities as educational tools that resist the increasingly dominant socio-temporal construction that “the pandemic is over” (Archie, 2022, para. 2). Such online communities also document the development of a living archive of sick, ill, and disabled knowledges, or what Piepzna-Samarasinha (2022b) calls the “work of our survival,” throughout the pandemic (para 15). Technological affordances, specifically remote opportunities to participate, have thus been invaluable to people with disabilities and chronic illnesses during the pandemic.

The obvious overlap between disabled *and* aging populations notwithstanding, separate bodies of scholarship have independently demonstrated the critical importance of art and technology for older adults and disabled people. Despite this, few studies have considered the value of digital technologies for artistic practice for people aging with longstanding disabilities and people first experiencing disability in later life—two unique yet overlapping intersections of aging and disability we refer to as *aging with/into disabilities* (Changfoot and Rice, 2020). Unlike the separate fields of aging and disability studies, which “often fail to recognize that people with disabilities age and that aging gives rise to disability,” the aging-disability nexus attends to the inter- and intra-sectional experiences of people aging *with* and *into* disabilities (Aubrecht et al., 2020, p. 7; Changfoot et al., 2022; Grenier et al., 2016; Korotchenko and Hurd Clarke, 2016; Lamb, 2015; McGrath et al., 2016; McFarland and Taylor, 2021). This paradigm challenges dominant aging discourses that define “successful aging” (Rowe and Kahn, 1997) as the prevention of disability and the maintenance of physical and cognitive function, and disability discourses, policies, and activisms that overlook age-related disabilities, such as age-related vision and mobility differences (Jonson and Larsson, 2009; McGrath et al., 2016). Put simply, the aging-disability nexus and by extension the concept of aging with/into disabilities conceptualizes disability as a *part of* rather than *distinct from* the aging experience (Aubrecht et al., 2020; Changfoot et al., 2022). To this end, we use the term disability to refer to disabilities experienced across the lifespan, including those developed in earlier life, those acquired in later life, and those anticipated to arrive in the future. In this usage, we reject the dominant convention of reductively associating disability with *young* bodyminds, who are predominantly assumed to exist outside of the normative linear life course (e.g., education, work, marriage, childrearing, etc.) and thus expected to have “no future,” and impairment—often configured as normal and expected—with aging or *old* bodyminds (Grenier et al., 2016; Changfoot and Rice, 2020; Kafer, 2013).

While few studies have considered the potential of digitally mediated creative spaces for older adults aging with/into disabilities, even fewer studies still approach this topic from a crip perspective that does *not* conceive of technology as a fix or cure to the “problem” of old age and/or disability. In one rare exception, Temple Jones et al. (2021) conducted a narrative literature review of disabled and aging people's experiences with technology and access design, or what they call “TechnoAccess” (p. 2). Building on the critical insights of crip technoscience and critical access studies (Chandler et al., 2023a,b; Hamraie, 2015, 2017, 2018; Hamraie and Fritsch, 2019), the concept of TechnoAccess rejects, or pushes *against* “technoableism,” the notion that technology can “solve” disability and thus “save” disabled people from disability itself (Shew, 2023). A TechoAccess approach recognizes instead how technologies may misfit with disabled and abled bodies and produce their own disabling effects (e.g., technologies such as diagnostic testing or the written word producing learning disabilities: see Rice et al., 2024). TechnoAccess prioritizes non-normative bodies, including disabled and aging embodiments, in technological development and scopes the “ongoing sociotechnical lives and corporeal realities of people's intersectional experiences, understanding that access is contingent upon social, structural, and technical barriers that are felt unevenly among users” (Temple Jones et al., 2021, p. 2). In their systematic review, the authors found that studies that implement storytelling methods, such as ethnography, most explicitly center “mad, disabled, and Deaf people's ‘corporeal attunements' in inquiry” (p. 9). Importantly, the authors note that, while the research they reviewed often reported on technology development *for* disabled and/or aging people, studies rarely worked *with* and their results were rarely drawn *with* and *by* disabled and aging people. To this end, our study aims to address a gap in the literature about age, disability, and technology, while responding directly to Temple Jones et al.'s (2021) imperative to “improve arts access in ways that centralize disabled, aging, and other marginalized people's multimodal experiences with technology” by eliciting and incorporating older adults' rich stories across all aspects of technology development and research design (p. 3).

Promoting a deep desire for social change, storytelling is an important and complementary approach to researching the needs and lives of underrepresented populations, including disabled and/or older adult populations (Chazan and Baldwin, 2021; Charise, 2022; Fraser, 2004; Nyboe and Drotner, 2008; Kaare and Lundby, 2008; Smith and Sparkes, 2007). Indeed, storytelling, particularly during the pandemic, played a significant role in shaping the identities and artistic expressions of older adults aging with/into disabilities, while opposing master COVID-related narratives, including “the pandemic is over” (Archie, 2022, para. 2). For instance, COVID in the House of Old uses wooden storytelling chairs to remember grief and outrage with the countless victims of Canada's eldercare system at the onset of the pandemic (Davies, 2022)—a time of mass eldercide, when older adults were often reduced to statistics in dominant news reporting on the effects of COVID-19 (Badone, 2021; Parekh and Underwood, 2020). Micro stories can challenge “generalizing and totalizing impulses” that disappear minoritized perspectives, and instead “tell small, situated stories that centralize the embeddedness of our embodiments” (Rice et al., 2022, p. 252), including those of older and disabled communities, which can trouble the violent necropolitical logics that undergirded global responses to and dominant narratives about the pandemic (Rice et al., 2022).

## Materials and methods

*Direct[Message]: Digital Access to Artistic Engagement* (*Direct[Message]*) is a collaborative, community based, arts-informed research project working within three mid-sized cities in Southwestern Ontario, Canada: London, Hamilton, and Guelph. In partnership with the Re•Vision Center for Art and Social Justice at the University of Guelph and the Schools of the Arts and Social Work at McMaster University, a Hamilton-based, community arts organization called *Centre[3] for Artistic and Social Practice* leads *Direct[Message]*. In keeping with the principles of TechnoAccess (Temple Jones et al., 2021), the project aims to make the arts more accessible and interactive *for* older adults through developing innovative digital technologies and environments *with/by* aging communities, especially those at the intersections of disability and other difference. To this end, *Direct[Message]* employs a community-based, co-design model that facilitates collaboration between older adult community members, artists, facilitators, academics, and staff from local community- and arts- based organizations and studios, including Museum London, VibraFusionLab, Cinematronics, and CreativeAge Network.

The project, which began near the end of 2019 and start of the pandemic, engages older adults from underrepresented communities in Southwestern Ontario. Shaped by the contours of a global pandemic (La Rose et al., 2022), Phase 1 (2019-2021) established strong and lasting collaborations with community partners through identifying barriers and supports that informed older adults' engagement with the arts. Phase 1 also involved the development of an easy-to-use keyboard and an easy-to-access web-based platform as prototypes to better support older adults' virtual arts engagement. Phase 2, the focus of this article, iteratively evaluated and developed these digital devices, including the easy-to-use keyboard with asynchronous art content and synchronous online art activities, that we thought would enhance older adults' access to and participation in the arts. This paper draws from interview data collected during Phase 2 of *Direct[Message]*.

The University of Guelph's Research Ethics Board (certificate number: 20-06-027) reviewed and approved the Phase 2 (2022) study in Spring 2022, while the initial study received ethical approval from McMaster's Research Ethics Board in 2019. Participants could participate if they identified as an older adult (i.e., 60 or older) or an E/elder (a title determined by cultural stature rather than age); experienced barriers (e.g., cultural, financial, physical, social, technological) to arts engagement; lived in or around London, Hamilton, or Guelph; and could understand and speak English. In keeping with the study's aim to prioritize underrepresented older adults, we used a “Recruitment Matrix” (Rice et al., 2020), a form of purposive sampling related to equity and inclusion, to recruit older adults with diverse embodiments and identities, including those from disabled, Two-Spirit, lesbian, gay, bisexual, trans, and/or queer (2SLGBTQ+), Black, Indigenous, and people of color (BIPOC), and newcomer, immigrant, and refugee (NIR) communities. Four 2SLGBTQ+, 11 BIPOC, and 13 NIR people participated. Several Arabic-speaking participants joined the study and chose a community translator to translate. Notably, nearly half (24) of participants self-identified as disabled, defined broadly to include mobility, sensory, and learning disabilities, chronic illness, neurodivergence (e.g., autism), mental health conditions (e.g., depression, anxiety, PTSD), and facial and physical differences (Table 1).

**Table 1:** Disability characteristics of study participants (*N* = 51).

**Participants identify as living with a disability**	
Yes	24 (47%)
No	26 (51%)
Prefer not to answer	1 (2%)
**Participants with disabilities identify in the following ways**	
D/deaf or hard of hearing	2 (4%)
Blind or visually impaired	1 (2%)
Living with a physical disability	8 (16%)
Autistic	1 (2%)
Neurodivergent	0 (0%)
Living with cognitive difference or challenges	3 (6%)
Living with a combination of disabilities	7 (14%)
Prefer to self-identify as [fill in the blank]	10 (20%)
Prefer not to answer	0 (0%)

During Phase 2, we invited older adult participants in the catchment cities to test a technology-based prototype (i.e., an easy-to-use keyboard) and a web-based platform (http://www.seniorsartlink.com), both of which were developed by *Direct[Message]* in collaboration with older community members to better support older adults' virtual engagement with the arts. We asked participants to test the keyboard and website by exploring at least one art-related activity offered by the platform. For example, participants could visit a virtual art gallery, attend a pre-recorded artist talk, follow step-by-step instructions to create an artwork, and/or explore relevant resources about digital literacy, such as instructions for creating a Zoom account. Ten older adults tested the keyboard/platform and participated in follow-up interviews about their experiences of using the keyboard to access art content/activities. We designed interviews to assess the potential of this type of digital technology to help older adults develop new digital skills, increase social interaction, and engage in artistic practice. These participants responded positively to the asynchronous content (i.e., the online platform) offered through the keyboard but explained that it was limited in that it did not connect them with other creative older adults. While participants generally expressed excitement about the wide diversity of digital content, including topics such as Deaf arts and the artistic exploration of the legacies of slavery and colonialism in Canada, they commented on how the keyboard's asynchronous format constrained social interaction. Put simply, participants wanted to create art synchronously (in real time) with others online, rather than creating art through asynchronous instruction. Based on the insights gleaned from the first round of data collection (i.e., the interviews in Phase 2), we focused on developing the digital platform's interactivity for further testing and paused keyboard development and testing.

To improve the digital platform's effectiveness for older adult communities, *Direct[Message]* in collaboration with older adult community representatives and artist-facilitators developed a series of online art activities for/by/with older adults. Forty-One older adults were invited to participate in one of six activities led by older adult artist-facilitators. In the summer of 2022, we offered the following online art activities: tribal [Sudanese cultural] doll-making, photography, an art crawl, drawing, digital storytelling, and collage. Each online art activity consisted of two parts: Part I oriented and prepared participants for the art activity and familiarized them with each other and the group's facilitators, researchers, and community consultants; and Part II provided context/instruction and engaged participants in an interactive art activity. Following art activity completion, we invited participants to take part in a 1–2-hour interview about their experiences of participating in the online art activity and other creative activities both online and in-person. Forty of the 41 participants who participated in the online art activities took part in a follow-up interview. In total, 51 older adults and E/elders living in Southwestern Ontario participated in Phase 2 of the study; 10 tested the keyboard, 41 tested the online art activities, and 50 participated in semi-structured interviews following their respective activities. Participants' first names are used to identify their individual responses and stories throughout. In instances where participants share the same first name and spelling, an initial was used to distinguish them (e.g., John F and John S). In instances where participants did not want to use their name, a pseudonym was assigned.

All interviews were conducted and recorded online, using a teleconferencing platform, by the first author between April and September of 2022, a time when federal and provincial COVID-19 protections (e.g., masking protocols and capacity limits) were being removed (Ontario Public Service, 2022). Notably, this context profoundly shaped the study's dynamics. Whereas previously, Canadian university research ethics boards were mandating virtual methods *only*, suddenly studies that sought to maintain social distancing practices to protect the health of researchers and participants alike and minimize the spread of COVID-19 were pressed to justify the use of virtual methods. Aside from contending with the changing expectations of research ethics boards, we also experienced internal disagreement among academic and community researchers about the online format, which as some highlighted would inevitably constrain the type (and success) of art creation and facilitation possible, as well as the social dynamics involved in a group setting. Ultimately aligning with a disability justice ethic of community care and interdependence (Berne et al., 2018; Piepzna-Samarasinha, 2018, 2022a), community and academic researchers collectively decided to continue conducting research activities online. The research team concluded that virtual research, though limiting for some older adults (e.g., with limited access to a stable internet connection and/or digital literacy skill-building opportunities), comprised the more responsive-to-difference ethical approach, given the ongoing spread of COVID-19 *and* its grave, disproportionate impact on disabled, aging, migrant, low-income, and racialized communities (Azeez et al., 2021; Garcia et al., 2021; Kamrul Islam and Hallstrom, 2023; Neely and Lopez, 2022)—many of the same communities at the center of our research.

We analyzed interview data using a combination of thematic and narrative analysis (Braun and Clarke, 2006, 2020; Reid et al., 2016). This approach allowed us to track “collective or shared meanings and experiences,” especially “*meaningful* pattern[s]” (emphasis added, Braun and Clarke, 2012, p. 57), across data, while attending to each individual's personal narrative (see Table 2 for a small selection of participants' diverse personal narratives, which are unique and overlapping). Our analysis is informed by critical disability studies and the theoretical insights of scholarship exploring the aging-disability nexus (Chandler et al., 2023a,b; Aubrecht et al., 2020), for as Braun and Clarke (2012) remind us, the production of themes is not a neutral or indifferent process; it is:

not like archeologists digging around, searching for the themes that lie hidden within the data, pre-existing the process of analysis. Rather, analysts are like sculptors, making choices about how to shape and craft their piece of stone (the “raw data”) into a work of art (the analysis). Like a piece of stone, the database provides the material base for analysis, and limits the possible end-product, but many different variations could be created when analysing the data. (p. 63)

**Table 2:** A small selection of stories from older adults aging with/into disabilities.

**Don**, a Black, Indigenous Two-Spirit person in his late 60s, is passionate about and an advocate for Indigenous issues, including the ongoing legacies of the Canadian Indian Residential School System, Two-Spirit culture, and Indigenous men's health. He enjoys using art, including painting, beadwork, and diamond pin art, to explore and celebrate his multiple identities as a double leg amputee and person aging with HIV. Don wants to visit local art galleries; however, as a wheelchair user, he regularly contends with environments that do not anticipate disabled people. Don is also restricted by the cost and location of such activities.
**Ida**, a woman in her late 60s living with post-traumatic stress disorder, enjoys the performing arts (e.g., tap dance) and textile arts (e.g., knitting). At the beginning of the pandemic, Ida learned how to upcycle old furniture and she participated in a few online arts classes (e.g., painting); however, she felt out of place because many of the other participants had advanced knowledge and skills. Despite having limited experience, Ida enjoys online options to participate in the visual arts, especially when navigating tough days with her mental health.
**Bernie**, a trans non-binary person in their late 70s who “transitioned late in life,” struggles to find activities that are not divided by binary notions of sex and gender (e.g., woman/man). During the height of pandemic lockdowns, they attended several programs and research activities that offered free arts-based content to seniors and/or people with mental health conditions. Living alone and grieving after the loss of their beloved cats, Bernie depended on these online activities for their social aspect, despite feeling “zoomed out” and stiff. Bernie generally seeks activities that explicitly display 2SLGBTQ+ inclusive symbols.
**Dorica**, a woman in her early 60s who has mobility issues and “a really big stomach,” uses a walker and a local paratransit service to get around and lives with a mental health condition that affects her sleep cycle—factors that shape her ability to participate in creative endeavors outside of the home. Prior to the pandemic, Dorica attended some creative in-person programs (e.g., coloring) primarily for the social aspect and enjoyment these activities provided. Dorica would like to visit art galleries in person; however, in such spaces, she needs access to spacious washrooms to accommodate both her body and walker and free or low-cost activities.
**Lynne**, an immigrant woman in her early 60s living with spinal disabilities and chronic pain, is a retired cake decorator and bakery owner. Living with an autoimmune condition, Lynne remains COVID-cautious because she caught COVID twice within the span of 6 months. As a chronically ill person, Lynne identifies as feeling “extremely lonely” and limited, especially as others around her started to return to in-person activities. She is interested in participating in art activities that help her combat loneliness and distract her from “looking out the window to see if anyone's come to see you.” The extensive time commitments and pacing of in-person activities, as well as the transportation to and cost of such activities, are challenging for her.
**Anthony**, an immigrant man aging into cognitive differences in his late 80s, has had a life-long passion to be an artist, but was pressured by his father to take up a more lucrative career in the trades instead. Once retired, Anthony returned to his passion and enrolled to take watercolor classes at the local library and became a Master Gardener. During COVID, Anthony attends online activities with the help of his wife, who increasingly provides greater assistance to him, including technological assistance.
**Radia**, a Black immigrant woman in her early 60s living with mobility and heart issues and recovering from cancer treatment, used to attend a weekly local knitting class, a treasured activity that reminds her of Sudan (her place of origin); however, Radia stopped attending when she experienced anti-Black racism. Radia wants to learn more about how to participate in online arts programming because getting around by foot is increasingly difficult and she has lots of free time; however, Radia worries that texting or writing in English may be a challenge.

Indeed, a commitment to valuing disabled and ill embodiments and knowledges, or “cripistemologies” (Johnson and McRuer, 2014), including those produced through the aging process, such as hearing, memory, and mobility differences, informed and animated our creation of themes. A commitment to disability justice, an intersectional and activist framework rooted in the experiences and perspectives of racialized, queer, trans, fat, and poor communities of disabled people also shaped the creation of themes (Berne et al., 2018; Clare, 1999; Lorde, 1997; Kafai, 2021; Piepzna-Samarasinha, 2018, 2022a; Schalk, 2022; Tidgwell and Shanouda, 2021). With the goal of attending to the specifics of, and advancing access and inclusion for, multiply oppressed disabled people, including those aging with/into disability, disability justice builds on the gains of the disability rights movement and critical disability studies by valuing and centering the leadership of those most affected by the outcomes. To this end, in prioritizing an intersectional lens, disability justice rejects biomedical and individualized understandings of disability and instead conceives of disability as a biological, social, and structural phenomenon informed by powerful intersecting structures (e.g., colonialism and racism).

Finally, in addition to our activist and social justice-seeking focus, our positionalities as authors, as intergenerational and interdisciplinary researchers and artist-facilitators engaged in researching, cultivating, and actively participating in aging and disability arts, also informed our analysis and the knowledge we produce. Our social positions as old/er, aging, disabled, racialized, queer, immigrant, ill, and allied co-authors inform our experiences with and understandings of aging and disability, the questions we ask, and concepts we explore in this study.

## Results

The current study investigated the stories that participants aging with/into disabilities told about their relationships to creativity and experiences accessing creative programming in-person and online. Drawing from 50 qualitative interviews with aging adults from un/under/represented communities, findings explore the intersections of older age and disability, including dynamics related to gender, sexuality, migration, size, race, and other differences, as these relate to their access to and enjoyment of creative spaces before, during, and “after” the COVID-19 pandemic.

### Existing creative opportunities are limited

Results suggest that existing creative/artistic opportunities are limited for older adults, both those experiencing disability for the first time in later life and those aging with long-standing disabilities developed earlier in life. Participants aging with/into differences described feeling unwelcome, discriminated against, and/or excluded when accessing artistic spaces and activities in their local communities. Specifically, participants reported how intersectional aspects of their nonnormative embodiments and identities, including but not limited to considerations of old age and disability, were neither adequately anticipated nor expected in existing local arts programming and thus rendered incongruous or “misfitting” (Garland-Thomson, 2011). The feminist materialist disability concept of misfitting “emphasizes the particularity of varying lived embodiments and avoids a theoretical generic disabled body” (p. 591), enabling analyses of multiple and intersecting identities and embodiments, including those uniquely experienced in later life *as well as* those experienced across the life course and compounded by the aging process.

Participants described how ageist and ableist stereotypical understandings about the interests and capacities of older adults resulted in few affordable opportunities specifically for seniors aging with/into disabilities. Lynne (see Table 2), for instance, recalled attending a creative activity designed for older adults where facilitators distributed children's coloring books and crayons, and instructed attendees to color. Thankful for the opportunity to meet and socialize with other older adults, Lynne said, “we were glad to do it, you know, it was lovely to sort of get together with people, it was lovely to do something artistic, but the level was childish.” “Because I'm over 60,” Lynne said, it is assumed “that I have regressed to three [years old].” Feeling infantilized by the type of creative activity and condescended to by the younger coordinators, Lynne expressed the need to feel “like an ordinary person having a lesson” and desired art programming that had “a little bit of an older person's point of view” to help minimize ageist and/or ableist sentiments. In another instance, Betty, a woman aging into cognitive difference, described aging out of the conventional art spaces/events she once enjoyed; she said, “I'm kind of a lost artist. As I get older, I don't have access, I don't have… I'm not surrounded by people who appreciate the same things that I do, and I feel isolated [sobbing].” Ageist and ableist attitudinal barriers, as well as a lack of creative opportunities tailored to the needs and interests of older adults who variously age into mobility, cognitive, and/or sensorial differences, produced environments and communities in which aging participants increasingly felt they could no longer fit into, both materially and socially.

Some aging and disabled participants described experiences of non-belonging or “misfitting” in local arts settings through discussions of race, migration, and language. Radia, a Black immigrant woman (see Table 2), stopped attending free knitting classes after she experienced anti-Black racist comments, and struggled to find other affordable, local activities once she left. Similarly, Dihnorath, an immigrant woman of color with a neurological condition that affects her gait, described how she felt she was being purposefully excluded from participating in some local musical and theatrical venues precisely *because of* her multiple differences. As a Columbian immigrant aging with mobility restrictions, Dihnorath explained that the stakes were higher for older immigrants because they have fewer opportunities to leave their homes, and thus fewer opportunities to practice English in ways that might foster their participation in the arts, unlike younger and presumably non-disabled immigrants. Ethnocentric language norms (e.g., Anglocentrism) worked in tandem with ableist and ageist structures to limit her opportunities to access the arts. In parallel, several Arabic-speaking newcomers, immigrants, and/or refugees reported language as a barrier to participating in local creative opportunities. For instance, a Black refugee woman who moved to Canada in 2020 during the pandemic's onset, Ibtisam E said, “in Lebanon it was easier, there was no language barrier.” Where she once ‘fit' into senior handiwork activities, she no longer could fit within a Canadian context due to a lack of linguistic diversity within local sewing/knitting classes. Stories of participants, especially racialized and migrant older adults aging with/into disabilities, reveal how racist, colonialist, and xenophobic assumptions structure who is assumed to physically and socially belong to (or fit into) creative spaces and, by extension, exclude “others” who fail to occupy those normative identities and embodiments, contributing to and intensifying experiences of exclusion related to age and disability.

Participants also described how they experienced misfitting related to gender and/or sexuality, which exacerbated their feelings of invisibility and alienation as older and disabled bodyminds. For example, Bernie (see Table 2), a non-binary person, felt they had no other option but to leave their gendered choir after transitioning, and now avoids creative activities that use gender exclusive language. Bernie explained:

I sing with [a gendered choir]. Well, I'm not going back there; I refuse to go back…people are asking. And I said, ‘no, I can't because it says [‘women' in the title].' ‘Yes, but they accept non-binary [people, someone claimed].' I said, ‘that's not the issue'. They'll still classify me as a woman; they'll say, ‘these *women*,' and ‘*she*'—there will always be ‘*she*' spoken. And I said, ‘No, I'm not [going unless]…it's a mixed choir or something…where I can make sure they know who I am. So, it's just trying to get away from things like that, where they [the organizers of creative programs] definitely make it male or female—nothing in between.

Similarly, Dana, another gender-variant participant described feeling alienated by both generational and intergenerational arts activities and events that were narrowly constrained by gendered expectations (e.g., feminine arts) and divided by gender. Feeling “invisible” as an aging Two-Spirited person in such spaces, Dana wanted to see arts programming and communities intentionally acknowledge gender variant artists/creatives and challenge gender binaries, saying, “I know that now there are even washrooms that are gender neutral. We don't [only] want washrooms; we want community that's gender neutral.” Additionally, Dana asserted that 2SLGBTQ+ communities in general are unseen or overlooked in the arts, maintaining, “we just are not targeted. We're not looking at Two-Spirited people, queer, trans, gay, bi people having art. And it's especially needed for these communities because we have a story to tell, and it needs to be shared.” These instances make plain how cis- and hetero-normative assumptions (i.e., oppressive ideologies that promote various normative ideas about aging, disability, gender, and sexuality, including sex and gender binaries—not to mention assumptions about which generations and/or age groups need and want non-binary spaces), structure creative programs in ways that can produce exclusionary dynamics for and amplify ageist and ableist notions about underrepresented older and disabled communities.

Participants embodying ability and size differences also reported experiencing physical, affective, and attitudinal obstacles when attempting to access existing arts spaces in the community. For instance, Don (see Table 1) detailed how environmental features, like stairs, actively exclude him from participating in creative local spaces:

I actually have to make sure it's wheelchair and handicapped accessible. If it isn't, that stops [me] from wanting to be part of [it]. There might be stairs going down. We had…[an event] and I couldn't attend [because] I had to stay upstairs because there's no way I can get downstairs to where the actual full event was housed. You know I'd like to [go] by myself and even listen to young guitar players doing recitals. I used to love doing that, but I can't get up and down from where I used to sit.

Recalling a time when he was younger and less disabled by the creative spaces he occupied, Don can no longer access the artistic communities he once fit neatly into because environments with only stairs anticipate and thus welcome people capable of ambulatory movement, actively excluding those who use assistive devices at any age. Relatedly, in rare instances when participants' stories detailed supportive experiences of accessing arts spaces/communities, these were generally presented in the past-tense, indicating that at one point in their lives, when they were younger and non-disabled, surrounding structures and relations enabled their participation. In another example, Dorica (see Table 2) described how narrow washrooms limit her ability to visit local art galleries as someone who has “a really big stomach” and uses an assistive device:

if it's not accessible then I can't use it…[and] if I do [visit a place] by myself I can't leave my walker unattended. And, plus, sometimes I can't use a washroom if it doesn't have a bar and enough space, I can't use it, physically use it, unfortunately.

Like the normatively exclusionary contours of stairs, narrow washroom stalls in art galleries assume that the enjoyment of art hinges on thin embodiments that *are* and *remain* unassisted by assistive devices, like walkers, wheelchairs, and scooters. Such examples illustrate how the material-discursive arrangements of a creative space/program can actively produce misfits and intensify experiences of alienation, non-belonging, and isolation felt by those aging with/into differences.

### Digital participation can promote access to creative engagement

Results from the current study also suggest that digital participation is “fitting” for older adults aging with/into disabilities and can foster and enhance access to creative engagement. Participants described virtual options as helpful and, in some instances, life-saving both before and during the pandemic, namely because remote options reduce physical barriers and eliminate distance/geography, expanding access. Moreover, those experiencing age-related mobility challenges (e.g., muscle weakness, joint tenderness, and swelling, etc.) and/or restricted access to community mobility (i.e., various forms of transportation that enable them to stay connected), described digital formats as promoting greater access to creative engagement because these can reduce a dependence on walking and getting around, especially in spaces that do not presume and thus anticipate people who use assistive devices. For instance, John F, a man aging into mobility differences, explained, “I'm having problems with walking and sometimes I have to use a walker. And going to Toronto, if you're going to see an exhibit then you've got to deal with subway stairs…so it can be more difficult.” Digital participation, he suggested, “would be very good for people like me who've just given up on going through the physical manifestations, wanting to watch it digitally.” Marianne, a woman who described “having a real hard time with [her] feet,” a change she attributed to getting older, reiterated this point: “I don't walk all that well anymore. So, it [virtual arts programming] was nice for me, like I said, to go around and not have to [walk]…to see the different things.” Radia (see Table 2) similarly explained that virtual arts programming was ideal for her: “I prefer if I have something to do from my home online… because my leg is still swollen.” Indeed, virtual options may support access for people like Margaret, who explained that walking with an arthritic knee to visit or move around an art gallery had become less of a possibility in later life, since even necessary outings, like getting groceries and attending medical appointments, had become taxing: “my mobility is restricted; it's painful for me to go any distance because of my knee.” As other participants indicated, digital options to participate (e.g., visiting a gallery or watching a performance) hold potential for those whom getting to and around materially inaccessible venues (e.g., with no elevators or accessible parking) is, perhaps, no longer realizable because the activity no longer fits their changing embodiment.

Virtual environments also allow older adults, including those without disabilities, to avoid the constraints of place and distance and enrich their creative engagement. For example, John S (a non-disabled man who finds virtual environments to be too “static”) said, “the benefit [however] continues to be that you meet with people in far flung areas…And if you're meeting with people across the globe, they're bringing experiences to the meeting that you wouldn't have in any other way.” Similarly, Victoria, a woman aging with mobility differences, explained, “online is an amazing tool right now…I'm grateful for it because I could do something over in Europe and I wouldn't have to go there. I don't like travelling, so [laughs]. I don't like going on the airplane, it's too claustrophobic, but I could if somebody set up a Zoom thing over there.” Digital arts enabled participants from all over, including places where resources and/or supports were more limited (e.g., towns and rural areas) to participate in activities that might otherwise be out of reach. As these responses suggest, online environments can bridge the physical gaps between spaces and, to an extent, democratize participation, benefiting both disabled and non-disabled older adults.

Participants also described digital participation in the arts as desirable because it reduces dependence on using personal, private, and public transportation for local travel, which can be unreliable, costly, dangerous, inaccessible, or simply inconvenient for those aging with and into disabilities:

I find it [online arts programming] very, very convenient. Because I don't have to travel anywhere to get there. (Jessica)

It's convenient. You don't have to travel; you don't have to park. You don't have to bring all your supplies with you. (Doris)

You don't have to go anywhere. You don't have to drive. You don't have to dress up and go to the theatre. (Bernard)

Zoom was excellent…because it took the barriers of geography out of [creative participation]. (Janice)

We've got everything here. We don't have to put everything in a bag and transport it to where we're going and set it all up on a desk. (Anthony)

Some participants also expressed wariness about the unfavorable conditions of public transit, which confine large crowds in spaces with limited or no COVID-19 measures left in place to protect them and support their travel to and from creative engagements. Increasingly dependent on walking to get around and thus limited by distance, Jerome described avoiding public transit; he said, “you got to be cautious, got to be careful. I don't ride the buses like I used to before…because there again in the buses, you're sitting next to other people who maybe are having the [COVID-19] infection. So, I do a lot of walking” (an activity that he described as becoming more difficult for him as he aged). Online, he concluded, is “more convenient.” For Jerome and his wife, and others like them, online activities reduced a dependence on unsafe public transit and enabled them to access activities that might not otherwise be accessible by foot. Thus, participants who could regularly and easily get around their communities as well as those whose mobility was constrained for various reasons (e.g., lacked vehicle access and/or someone to assist, had caregiving and/or work responsibilities, etc.) similarly described a fit between digital participation and their older and/or disabled embodiments.

Participants also described becoming increasingly wary of and altogether avoiding unfavorable conditions as they age with and into disabilities. Less likely to drive or walk in the snow and/or at night, participants described virtual options as supporting their participation generally, and arts engagement, specifically. Nancy, for instance, said, “in the winter, you got to go out and the weather's bad, the roads are bad, the sidewalks are bad and that just puts you off altogether. So, having something online like this, it's a great thing to offer.” In parallel, Lynne preferred online options, explaining “if it was snowing or raining or whatever, I couldn't go. I can't risk falling because of the state of my spine, so I don't go out when it's icy.” Other weather events also posed a threat to older adults' creative engagement. Bernardine explained, “some places are not easy to reach, right? In summer it's particularly hard on hot days to walk. It takes 45 minutes for us to walk from [home] to [the local arts venue].”

While some participants described virtual formats as imperfect substitutes and incompatible with in-person arts environments, many others, especially those constantly or gradually misfitting with creative communities and spaces, detailed how virtual environments supported and expanded their participation in the arts. In these instances, participants understood online environments as safer for older adults with existing and recently acquired disabilities and health concerns, and for those actively trying to avoid losing vitality as a consequence of COVID-19. For instance, several participants reported wanting virtual options to negotiate the risks of the pandemic, including Sheila, a non-disabled woman who had regretted a recent visit to a crowded indoor art gallery:

People were too close to each other. There was no six feet apart and everybody's wearing masks and you're like, ‘Okay, don't stand too close to me', and…if I had been able to watch that online I would've enjoyed it a lot more. And tours of galleries like online, I'd like that.

Despite some remaining pandemic controls to protect the public (e.g., community masking), which have since been rescinded—regardless of the continued circulation of the SARS-CoV-2 virus and the documented threat of the virus's mass disabling effects (Davis et al., 2023; Amisi, 2024), Sheila felt anxious negotiating indoor spaces during the pandemic. Several non-disabled older participants, though equally exhausted with lockdowns and other pandemic controls (e.g., proof of vaccination rules and capacity limits), expressed frustration and concern with removal of pandemic controls in public settings—a decision that, many disability justice advocates argue, makes public space more dangerous and less accessible for everyone, generally, but ill, disabled, and/or older communities, particularly (Piepzna-Samarasinha, 2022b; Rajkumar, 2022; Adler-Bolton, 2023; Amisi, 2024). For instance, Rob, a non-disabled man of colour, explained that he had not yet returned to his favorite creative program, despite its recommencement, because, he said, “I'm not feeling confident around the controls around COVID these days with the numbers going up and up.”

Participants aging with disabilities (i.e., those acquired earlier in life) also described digital environments as safer and thus preferable. For instance, Ida (see Table 2), a woman living with post-traumatic stress disorder, explained how virtual formats enabled her to “talk freely about [her] past” in a creative storytelling workshop. The digital format “with the little screen,” she said, “helped me in…that I could tell my story in front of men.” Alluding to a relationship with gender-based violence, Ida explained, “because of my past, I tend not to search out [men] for an audience.” The online environment, which afforded her the comfort of her home and physical distance from men, “made it seem safer” to present her story to a mixed group. Additionally, she explained, “because I have PTSD… some days, I just don't feel like getting out of bed. It's quite limited what I can do. And so…I love doing things online because I can stay at home, [and] that helps with those days that I'm not feeling that terrific.” Similarly, Janet, a woman with sensorial and mental health differences, described online arts spaces as “really important” for herself and others who were aging into disabilities that restricted their movement:

After my car accident, I had balance problems and vision problems and a ton of vertigo, so going out when you can't walk, or you might fall over, doesn't feel safe. And I have friends who have now developed Parkinson's, ALS; they don't get out anymore at all. So, the online stuff just feels so much more accessible.

Janet described digital spaces as essential, explaining, “It [a virtual environment] just opens up my world.” In another instance, Jo-Ann, a woman who reported having social anxieties, said she found the online experience of both facilitating and receiving art instruction as “a lot easier.” She explained that in-person, in a big room with multiple people, “you get kind of nervous,” whereas “when you're just online, you're just seeing one person most of the time…so you feel a little bit more relaxed and at ease to do it.” Digital environments shrink expansive physical spaces and the social threats they hold, which for both Ida and Jo-Ann produced a comfortable “fit” and expanded their capacity to participate fully. Highlighting another benefit of the online experience, Jo-Ann said, “I actually like learning online now better than even going to a studio because you can pause it [a recording] and go back.” The ease in which online videoconferencing platforms, like Zoom, enable audio and video-recordings, benefited older adult participants, especially those with learning and other disabilities and/or illnesses that could suddenly, yet chronically, interrupt their ability to participate in-person. Dana, for instance, said, “online is ideal because anything I would register for, they would have a replay and that would be so ideal” for “when I'm in pain and then I need to go into therapy.” Also beneficial for those with learning differences, Penny said, “for me, the way I learn and the way I process my learning experiences is I listen, I mull things over, munch my way through it and then I actually probably need to hear it again. So, for me, online works really well because I can back that video up.”

Finally, some participants conceptualized virtual environments as beneficial because they afforded them permission to comfortably sidestep certain taken-for-granted expectations of meeting in a physical environment, such as remaining seated or attentive for long periods of time. Jane, a woman living with chronic pain and learning disabilities, described how some online environments enabled a passive or relaxed form of participation that she found fit her bodymind. She explained, “I do like online, too, where you like turn off your video, go make a cup of tea or do something and…you can still listen, but you don't have to be present for the whole time. You can do some self-care.” Similar to relaxed performance (LaMarre et al., 2021), where the atmosphere and unspoken “rules” or “norms” of a creative environment (e.g., a theater) are relaxed for people with sensory and cognitive differences, online spaces, with features to adjust volume and turn cameras on and off, have the potential to promote relaxed participation.

### Digital arts that multiplied *then* are *now* disappearing

Results from the current study also indicate that older adults benefited from the flourishing of digital arts during pandemic time. Participants described experiencing an explosion of free and affordable virtual activities during the first two years of the pandemic, including arts-based research activities that governments in Canada supported as part of COVID-19-related funding allocations. For instance, Janet, a participant who used virtual options both before and during the pandemic, reported witnessing virtual opportunities to view art increase substantively during pandemic time; she explained, “It's when most of these galleries opened up and put all their artwork online.” In some instances, participants described having unprecedented access to exhibitions in otherwise out-of-reach venues and artistic skill-building opportunities/instruction that programmers previously made available in only particular cities or neighborhoods, where they had registered demand for such activities. However, participants' responses indicated that whilst those living outside major centers enjoyed newfound access to these opportunities, they soon realized that they could not count on these life- and culture-expanding access affordances to continue.

Indeed, although digital arts multiplied during the first couple of years of the pandemic, these opportunities started to disappear as “pandemic time” began to wane, despite the predicted threat and felt consequences of new variants. Participants described experiencing the loss of digital opportunities, arts-based and otherwise, as the pandemic was slowly, at the time of interviewing, being discursively constructed as ending (Archie, 2022, para. 2). Bernardine, for instance, explained that her opportunities to participate in creative programming were decreasing because a knitting class she was once able to attend online was, at the time of the interview, returning to in-person participation exclusively. When the class moved back to in-person, Bernardine could no longer attend as often because of distance, cost, lack of access to a vehicle, and the danger public transportation posed to her health. Relatedly, lamenting the transition from online to in-person, Lynne described the value of online virtual arts programming and emphasized the long-lasting potential it holds for the health and wellbeing of older and disabled communities, especially lonely and/or isolated populations, both within and outside of pandemic time:

If I could wave a magic wand and decide what [the program] should do I would say, ‘don't get rid of the virtual things because there were, and I include myself in this, some lonely people who looked forward to it at least once a week, seeing some faces and having a chat, right?' So, that was very important, that aspect of it. I don't get out much and some of the seniors that joined the group don't get out at all, so for them going to the in-person sessions is not that practical and especially with the colder weather…Or if it's raining or, you know, if they're not well that day or whatever, you know, they won't be going at all and then it will be two months until they have some interaction with somebody.

Similarly, Katherine described how the conditions of the pandemic produced possibilities for her as a person aging into physical disabilities, noting the effect the return to “the way it was” would have on people who were disabled prior to the pandemic:

The pandemic really stood out for me, as someone who was becoming more physically limited, at how much [was] accessible; things were being made for people. And now that everything is moving back—most things are moving back to in-person experiences—I'm sort of sitting here going, “well, what do I do with myself?” And it really hits home for people who have accessibility issues, and mobility issues, [for whom] this was their whole life before the pandemic, and they were given a bit of a lease on life through the pandemic, and now they're being shut out of spaces. I see this, particularly with the two [craft/art groups] I'm a member of—about how people have been kind of desperate to get back to in-person meetings. And a small number of people [are] saying, ‘the only reason I could come to every meeting was because it was online'. So, it's been an interesting time, I think, to think about these things. But I'm pretty sure we're going to just try to go back to…the way it was.

As Katherine indicates, a return to “normal” creates the real risk that many disabled people, including those aging with disabilities, will lose critical access to creative communities that, for perhaps the first time in their lives, produced a “fit,” rather than a “misfit.” Even when participants, especially those who faced few mobility barriers, expressed a desire to return to preferred in-person creative events, they acknowledged people's differing perspectives and the continued need for virtual options for older and disabled communities rendered less mobile by inaccessible infrastructures, including their future bodyminds, which (failing a transformation of the built environment) they imagined as less physically mobile and more confined to the home. For example, explaining that online activities were less relevant for him, given his ability to get around with the assistance of his wife, Anthony said, “we're not shut in.” Nonetheless, he described value in having such options because, as he explained, “one day, I'm going to be shut in. I don't drive anymore, so it's [my wife's responsibility]—if ever [my wife] gives up driving, I don't know what we're going to do.” In another instance, Penny, a passionate advocate for aging arts and artists, noted that among her older adult membership there was a desire to remain online for reasons that pertained to and exceeded the pandemic and COVID caution:

I got resounding ‘yeses' to go back to Zoom. They [older adults] don't want to worry about, “Is it [the class] on today?” “Do I come today?” “It's snowing in [the city], should I come?” You know, “there's going to be an ice storm at 4 o'clock, will we be done by then?” You know, they don't want to worry about that. I get that. So…we'll probably go back to Zoom. And they seem to be quite happy with that.

Reiterating the significance of transportation infrastructures and weather events to the community mobility and creative engagement of older and disabled communities, both Anthony's and Penny's commentaries point to a collective demand for digital options to engage in the arts both during and after the pandemic, now and in the future, and for others and their own older embodiments, which they imagined as becoming more disabled in time.

## Discussion

Results show that older adults aging with/into disabilities in Southwestern Ontario express an overwhelming desire and even urgent need to access interactive arts programming from the relatively safe spaces of their homes both within and outside pandemic time. As the normative world pushed for a return to ableist normative life in 2022, a year marked by “severe” rates of the highly infectious Omicron variant and the loss of effective public measures, such as community masking and widely available testing (Ontario Public Service, 2022; Public Health Ontario, 2022), participants described the need for continued access to creative and social participation via remote options that sidestepped socially exclusive (e.g., racist and cissexist) and physically inaccessible (i.e., ableist, ageist, and sizeist) spaces. Participants also described experiencing disparities related to in-person and virtual art activities for older adults in Southwestern Ontario. Prior to the pandemic, underrepresented groups of older adults, including those with disabilities, experienced “misfitting” or non-belonging in creative spaces that were designed with a normative user, or “normate template” in mind (Hamraie, 2017, p. 19; Garland-Thomson, 2011). According to critical access scholar Aimi Hamraie, far from an “average” or “universal” user, this template presumes and is made in the image of a “white, European, nondisabled, youthful, and often masculine figure whose features remain unmarked” (p. 20). Neither neutral nor universal, spaces and environments created with a “normate template” produce a seamless or seemingly natural “fit” between specific users and their environments. Those whose bodies and ways of being deviate from this norm (e.g., older people, people with disabilities, and people of color) will experience a “misfit” with an otherwise neutral appearing environment, reifying the notion that particular bodies or subjects are the “problem” (Garland-Thomson, 2011). Participants described how the design and delivery of creative in-person environments were, prior to the pandemic, exclusionary primarily because they presumed and anticipated specific unmarked bodyminds (e.g., white, anglophone, thin, cisgender, heterosexual, ambulatory, etc.). For instance, non-binary and Two-Spirit older adults aging with/into disabilities experienced “misfitting” in creative activities that presume binary and Western gender norms (and thus anticipate cisgender, heterosexual, and settler users), while older adults who use assistive devices, like wheelchairs and walkers, experienced “misfitting” in artistic environments that privilege normative bodies (and thus anticipate thin and nondisabled users). The inaccessible social and built environments of in-person artistic communities, and the limited options for traveling to and from such places, foreclosed in-person participation for many participants who do not reflect the “normate template.” And, if cultural and social service organizations had created online opportunities for older adults to participate in the arts during “pandemic time,” participants described—often with sadness and despair—how these opportunities were temporary and/or stopped as pressures to return to normal mounted.

These findings indicate a need for increased investment in digital arts programming, as well as digital skill-building opportunities, for older adults aging with/into disabilities. Celebrated for their life-sustaining potential, such programs, as one participant explained, “got a lot of us [older adults and people with disabilities] through the pandemic” (Lynne). They provided opportunities for various older adult communities to socialize, develop digital and artistic skills, build community with others, and care for one another. Although generally associated with (younger) crip and disabled access-making communities (Gotkin and Hamraie, 2024; Hamraie and williams, 2023; Johnson et al., 2024), remote access offered many participants aging with/into disabilities the chance to experience the value of and glimpse the potential for digital participation both during and beyond pandemic time. For older adults considered “vulnerable” to COVID-19, those with mobility and/or mental health disabilities, and those with caretaking duties, online opportunities allowed them to participate comfortably from their homes. Digital forms of arts engagement may be particularly promising for those aging *into* disabilities (i.e., those who were non-disabled for most of their lives and are now experiencing ableist exclusion). Remote options and communities may possibly provide a sense of belonging to those newly coming into a disability and/or disabled identification and consciousness, potentially offsetting the shame, guilt, and alienation experienced when aging in an ageist and ableist culture that propounds the imperative to age “well” or “successfully;” that is, without cognitive or bodily change and dependence, which are predominately conceptualized as markers of decline, failure, and weakness across the life course (Aubrecht et al., 2020; Grenier et al., 2016; Gullette, 2004; Lamb, 2015; Leahy, 2023; McFarland and Taylor, 2021). For as some have demonstrated, there is reluctance from many older adults to align with disability in part due to the triumph of “successful aging” paradigms (Leahy, 2023; McGrath et al., 2016; Oldman, 2002).

Indeed, remote access holds possibility for many marginalized groups outside of a pandemic-context, *as well as* for those who are disabled by the presence of COVID-19 and a lack of federal and provincial COVID-19 measures (e.g., community masking) to support their in-person participation. However, despite the value digital arts hold for multiple communities of aging and disabled people, hegemonic orders arguably only ever intended the online pivot to comprise a temporary “emergency” response that, as Ellcessor (2022) demonstrates, governments and corporate interests designed to provide protective access to life/work for a young, healthy, nondisabled majority. Older adult participants found use for technology, specifically remote access, in ways that both fit comfortably within and exceeded the contours of pandemic time. Within pandemic time, some disabled older adult participants experienced what some call “crip utopias” and “crip hope,” whereby disabled communities “hope that some of our adapted practices taken from disability culture, including access practices, will stick around outside of the disability community after COVID time” (Ignagni et al., 2020). In particular, our findings point to the value crip remote access practices hold for older adults aging with or into disabilities. The return to normal and the attendant “snapping back” to in-person creative activities both increases aging, disabled, and ill people's risk of contracting the virus and excludes them/us from environments and spaces that were only ever constructed with a “normate” user in mind. In important ways, older adults' creative engagement with accessible remote access environments takes a lead from crip culture in offering another pathway that nonnormatively embodied people can take to repurpose technology for their own anti-ableist and -ageist ends (Rice et al., 2024). In this case, by proliferating material (affective, sensorial) and social connections between bodies and worlds, *Direct[Message]* expanded possibilities for aging and disabled embodiment and life.

As many critical disability scholars, activists, and artists have demonstrated, non-disabled people directly benefited from the knowledges and strategies of disabled communities during the COVID-19 pandemic (Chandler et al., 2023a; Johnson et al., 2024), despite the violent exclusion of disabled people and their needs from national health emergency preparedness and planning (Pearce et al., 2022). The findings from this study indicate that underrepresented communities of older adults may be one such group that has benefited from remote access during pandemic time and would continue to benefit from such access after pandemic time. An AGE UK report (2018) found that having access to wealth and transportation, especially a vehicle, is strongly associated with older adults' creative and cultural participation. They recommend that arts organizations “take transport into account in their programming, and consider how they can get their activities out into more accessible locations, including care homes” (AGE UK, 2018, p.12). Our findings indicate that accessing creative activities and content virtually, comfortably in the home, may be one desirable possibility, which reduces a dependence on poor transportation or stressful travel (i.e., driving at night, in poor weather, and/or for long distances) for supporting older adults' engagement in creative and cultural programming. Fluharty et al. (2021) similarly suggest that the digitalization of cultural and artistic events, like museum tours, may be one way in which barriers, like cost of participation and dependence on transportation, may be reasonably mitigated for those navigating exclusionary systems. However, digitalization can also, they surmise, “bring further age related inequalities among those with low digital literacy” (Fluharty et al., 2021, p. 13).

Indeed, digital technologies and environments are not free of power dynamics, nor social inequalities, and can reinforce harmful and exclusionary biases and structures (Zheng and Walsham, 2021; Jonsson et al., 2023). As many studies have indicated, including some of our own (Hand et al., 2024; La Rose et al., 2022), remote participation using digital technologies is not without its own set of barriers for older adult and/or disabled community members. As Charise (2022) reminds, “it is important to challenge assumptions about access to the internet, which is clearly affected by class, socio-economic status and, indeed, age,” while recognizing the harmful effects of the very existence of digital communication tools (e.g., search engines) and infrastructures, which literally invade and harm Indigenous territories and communities, including disabled people, E/elders, and those who will hopefully become old (p. 241). Therefore, it is vital to recognize how forms of structural disadvantage, like colonialism and poverty, also affect aging, disability, and digital arts access. Further, even with reliable access to the internet, older adults and disabled communities can face challenges navigating videoconferencing technology and etiquette. For instance, Hand et al. (2024) found that out-of-date software and digital devices, misunderstandings regarding the conventions of videoconferencing (e.g., muting microphones to reduce background noise), and inadvertent cross-talk and trouble with turn-taking posed problems for older adults' remote participation. As well, Clayton et al. (2023) found that inaccessible software, including videoconferencing platforms, and the pace in which technology and accessibility equipment evolves and older and disabled bodyminds change, to be challenging for older and disabled people using technology during the pandemic. It is also necessary to recognize that some older adults will never engage with digital technologies (Clayton et al., 2023). Thus, although the current study found strong evidence for remote creative participation, there cannot be a “one size fits all approach” to the creative and social participation of older adults aging with/into disabilities because bodyminds are diverse and have diverse needs. Indeed, crip and disabled practices and ontologies—not unlike those experienced in later life—remind us that an accessible world is possible, and that access is “an iterative and co-designed process” (Chandler et al., 2021, p. 230; Chandler et al., 2023a,b). To this end, a hybrid model that offers multiple forms of communication and participation, including remote (e.g., mail, phone, digital) and in-person, might better support more older adult and disabled people.

Aside from presenting an evident need for remote options to support older adults' artistic engagement, the current study's findings advance scholarship exploring the aging-disability nexus, art, and technology in the following three ways. First, building on critical age studies literature that exposes and challenges the ableism and ageism of successful aging initiatives and scholarship, the findings extend crip, critical disability, and TechnoAccess insights to the study of aging and later life in ways that do not devalue nor seek to “correct” the so-called “problems” of aging and disability through engagement with technology and/or art. Second, the findings offer valuable qualitative data about aging and disabled people's participation in artistic activities and experiences with technology. Attending to some of the gaps identified by Chacur et al. (2022) in their scoping review of the existing literature on older adults' artistic engagement, the current study contributes important knowledge about some of the attitudinal, material, environmental, and structural barriers older adults aging with/into disabilities experience when attempting to access the arts. As they note, such practical knowledge is necessary to successfully promote older adults' artistic participation. The current study also privileges the experiences and voices of older adults and centers the most underrepresented voices aging with/into disabilities. Chacur et al. (2022) found that “other minority groups such as older people belonging to ethnic or racial minorities, older immigrants, older adults with disability, older members of the LGBT community, or the oldest-old people are underrepresented in this topic research, if not absent” (p. 940). This study responds directly to this gap, offering diverse qualitative data. Finally, the current study complicates dominant ageist and ableist notions that older adults and disabled people are digitally incompetent and/or unwilling to learn new technologies and invites crip access-making communities and spaces to consider and take seriously older adults as a community who may also require or want digital access.

Importantly, these findings are limited by a sample population that overwhelmingly identified as “very” or “somewhat” confident using digital devices (e.g., laptops, smart phones, tablets) and digital platforms (e.g., Facebook and YouTube). Most participants in the current study reported using digital devices and platforms daily, indicating relatively moderate to high digital literacy levels. Notably, all digital skill levels were encouraged to participate, technical coaching and phone and masked in-person support were offered, and internet-enabled devices were loaned to those who lacked the digital tools and/or knowledge necessary to participate. Only three participants loaned iPads and four required additional technical support (e.g., learning how to use a new type of technology, creating a Zoom account, and/or recovering a lost password). To this end, research with older adults with low to no digital literacy would allow for better understanding of some of the barriers to and supports for remote access, generally, and digital arts, in particular.

Finally, to better meet the needs of a growing number of older adults aging with/into disabilities in Canada (and beyond) and increase the effectiveness of cultural and artistic resources and programs, our findings indicate the importance of meaningfully engaging with arts and culture programmers (e.g., art museums, libraries, and seniors' programming, etc.) and end-users. Findings suggest that prioritizing end-user experiences, including those of older adults, disabled people, and members of BIPOC, 2SLGBTQ+, and immigrant communities, can help providers better understand how to equitably anticipate and serve diverse user communities. Finally, we recommend and advocate for the inclusion of older adults aging with/into disabilities throughout all levels of decision-making regarding artistic and cultural policy development and program implementation.

## Data Availability

The original contributions presented in the study are included in the article/supplementary material, further inquiries can be directed to the corresponding author.
